# What is the fate of scientific abstracts? The publication rates of abstracts presented at the 7th National Congress of Gynecology and Obstetrics

**DOI:** 10.4274/tjod.77785

**Published:** 2015-03-15

**Authors:** Gülçin Şahin Ersoy, Deniz Öztekin, Ayşe Gül Kebapçılar, Tutku Gürbüz

**Affiliations:** 1 Marmara University Pendik Research and Education Hospital, İstanbul, Turkey; 2 Tepecik Research and Education Hospital, İzmir, Turkey; 3 Selçuk University Faculty of Medicine, Konya, Turkey; 4 Ardahan State Hospital, Ardahan, Turkey

**Keywords:** meeting abstracts, Congresses, manuscripts, unpublished works

## Abstract

**Objective:**

Oral and poster presentations held at national and international congresses are recognized as valuable tools for sharing current scientific data and experience among physicians. However, a large proportion of these works fail to be published in scientific journals. We have designed a study to identify the publication rate of presentations held at the 7^th^ National Congress of Obstetrics and Gynecology in 2009.

**Materials and Methods:**

A systematic search of databases was performed using author names and key words from the abstract title to locate publications in peer-reviewed journals corresponding to the presentations held at the 7^th^ National Congress of Obstetrics and Gynecology. Information regarding mode of presentation, topic, type of affiliation, name and impact factor of the scientific journal, change in author names and time elapsed between presentation and publication were recorded and analyzed statistically.

**Results:**

Of 243 abstracts that were presented at the congress, 45 papers (18.5%) were published in international peer-reviewed journals, whereas 39 (16%) were published in national journals. The mean time to publication was 17±2 months for international and 11±4 months for national journals (p=0.102). The international publication rate of oral presentations was significantly higher than that of poster presentations (50% vs. 16.2%; p<0.03). The manuscripts were published in a total of 21 journals, with four journals accounting for 49% of the publications. The comparison of the publication rates of the universities with other institutions has yielded no significant difference.

**Conclusion:**

Alltough a significant proportion of the abstracts presented in the 7^th^ National Gynecology and Obstetrics Congress have been succesfully converted to publication overall, only a limited percentage of all abstracts have been published in international peer-reviewed journals. The relatively higher conversion to international publication rate of the oral presentations show that they are of higher interest and clinical relevance.

## INTRODUCTION

Presentations (meeting abstracts) are short summaries of investigations designed to be presented in scientific congresses. They facilitate sharing of scientific studies with other colleagues and the generation of potentially useful feed-backs^(1)^. The conversion of these presentations to publications in well-respected national and international journals is an essential step towards increasing the overall number of researchers who can access the information provided in these meeting abstracts without attending the actual congress. The Cochrane review published in 2007 has revealed 31% conversion rate of congress presentations to publications^(2)^. According to this review, most of the studies presented in meetings could not be transformed to publication and, therefore, fail to contribute to the scientific literature.

The publication rates of abstracts presented in obstetrics and gynecology (Ob/Gyn) meetings were reported between 38% and 52% in previous studies^(1,3)^. However, to the best of our knowledge, the presentations held in a national gynecology and obstetrics congress in Turkey have not been analyzed before. Each year approximately 300 abstracts are presented in the annually held congress organized by the Turkish Association of Gynecology and Obstetrics (TJOD), with a broad participation by Ob/Gyn specialists. The designation of the publication rate of these presentations in national as well as in international journals can be regarded as an important and objective measure revealing the actual level of contribution exerted by the Turkish researchers to the overall scientific knowledge.

The aim of this study, therefore, was the identification of the actual publication rates of oral and poster presentations held at the 7^th^ National Gynecology and Obstetrics Congress of TJOD in May 2009 and the analysis of factors suspected to play a role in this conversion process.

## MATERIALS AND METHODS

All poster and oral presentations held at the 7. National Gynecology and Obstetrics Congress in May 2009 were included in our cross-sectional study. All data related to the presentations have been obtained through the official web-site of TJOD at http://www.tjoddergisi.org. PubMed (http://www.ncbi.nlm.nih.gov), Google Scholar (http://scholar.google.com.tr), Turkish National Database (http://uvt.ulakbim.gov.tr) and Turkish Citation Index (http://www.atifdizini.com). Literature searches were performed to determine conversion rates. The search in databases has been started in June 20, 2014 and completed in August 01, 2014. As the first step the title of the presentation has been scanned in all databases for a possible cross-match. If a match was not found, a key word selected from the abstract title has been combined with the name of one of the authors, starting with the first author. In case no match has been found, the publications of each author have been individually scanned as the last resort. Following the detection of a publication matching one of the presentations in question, the summary of the article has been thoroughly analyzed and the match-up has been confirmed. If there was any doubt regarding whether an article matched the abstract, all researchers of the study were asked for individual evaluation and a consensus has been reached. When a match was found, the full title of the published article, the contributing authors and their affiliation, the journal and its impact factor, as well as the date of publication were entered into the database. In case more than one affiliated institutions were present for a publication, only the institution of the first author has been taken under consideration. The publication dates were recorded in month and year; if the data included two consecutive months, the first one is selected. If only year info could be found, the month of publication has been designated as June.

The assembled data have been analyzed for (1) the publication rates, (2) the average duration for publication, (3) author’s affiliated institutions, (4) journals and their impact factors.

Statistical analyses were performed using the Statistical Package for the Social Sciences( SPSS) version 17 program (SPSS Inc., Chicago, IL, United States of America). The variables were investigated using the Kolmogorov-Smirnov test to determine whether or not they are normally distributed. The data with normal distribution were analyzed with an independent samples T-test whereas the data without normal distribution were evaluated using the Mann-Whitney U test. The categorical variables were analyzed with the Fisher’s Exact test. Numerical variables were reported using mean and standard deviation; categorical variables were presented using percentage. A p-value of less than 0.05 was considered to show a statistically significant result.

## RESULTS

Of the 243 meeting abstracts presented at the 7th TJOD National Gynecology and Obstetrics Congress 84 (34.5%) were successfully published; 45 (18.5%) of them were published in peer-reviewed international journals and 39 (16%) in peer-reviewed national journals. The comparison of all abstracts according to their type of presentation has revealed that 50% (n=8) of the oral presentations and 16.2% (n=37) of the posters were published in international journals (p=0.03; odds ratio=5.33). The publication ratios in national journals were 6% (n=1) and 16.7% (n=38), respectively.

The overall mean time to publication was 17±2 months for international journals with an upper limit of 60 months. Among the abstracts published in the international journals, the mean time to publication was calculated as 18±2 months for posters and 16±6 months for oral presentations (p=0.533). Almost 84% of all abstracts accepted for publication were published in the first three years following the congress; on the other hand, 4% were published after 4 years have passed (Table 1). Among the abstracts published in the national journals the overall mean time to publication was 11±4 months; it was further calculated as 13 months and 11 months for oral presentations and posters, respectively (p=0.741). There was no statistically significant difference between the international and national journals regarding the mean time to publication (p=0.102). Seven of the studies were first published and then presented later in the congress.

The publications comprise 21 different international journals and 49% of these articles were covered by 4 journals alone: International Journal of Gynecology Obstetrics, International Journal of Gynecological Cancer, European Journal of Gynecological Oncology, and Clinical and Experimental Obstetrics and Gynecology (Table 1). The mean impact factor of these 21 journals were calculated as 1.572±0.126 (min=0.380, max=4.174). The comparison of the presentation types for journal impact factor averages did not reveal any statistically significant difference (Table 2).

The analysis of the abstracts according to the type of institution has revealed that 135 (55.6%), 86 (35.4%), 10 (4.9%) and 9 (4.1) abstracts were prepared by the university hospitals, education and research hospitals, private institutions and state hospitals, respectively. Overall in each subcategory, 29 out of 135 (21.5%), 13 out of 86 (15.1%), 2 out of 10 (20%) and 1 out of 9 (11.1%) abstracts were successfully published in international journals. Although the rate of conversion to an international publication was found to be relatively higher for university hospitals, the comparison of the institution types for publication ratios did not reveal a statistically significant difference (p=0.184). The rate of conversion to a national publication was determined as 13.3%, 15.1%, 20% and 66.6% for the university hospitals, education and research hospitals, private institutions and state hospitals, respectively.

The meeting abstracts were gathered under 6 headings as general gynecology, general obstetrics, gynecological oncology, perinatology and maternal-fetal medicine, urogynecology, and reproductive endocrinology and infertility. Among these headings, most abstracts belonged to general gynecology (n=65, 26.7%), whereas the highest conversion rate to international publications has been observed under the gynecological oncology heading (Table 3). The conversion rates to national publications has been found for general gynecology, general obstetrics, gynecological oncology, perinatology and maternal-fetal medicine, reproductive endocrinology and infertility and urogynecology as 23% (n=23), 20.5% (n=8), 9.5% (n=2), 8.1% (n=4), 9.4% (5) and 31.2% (n=5), respectively.

The analysis of the authors of the published abstracts has demonstrated that in 71.1% of abstracts published in international journals, some changes have been made in author names. Among them, 22.2% had an addition of a new author name, 17.8% had a deletion of an author name and 31.1% had both changes simultaneously. Similarly, a change in author names has occured in 74.3% of the national publications. In this subgroup 30.7% had an addition of a new author name, 15.3% had a deletion of an author name and 28.2% had both changes simultaneously. Thus, overall 72.6% of all presentations published in international and national journals have undergone a change in author names during the conversion process.

## DISCUSSION

A publication ratio of 34.5% has been found for the meeting abstracts presented in the 7^th^ TJOD National Gynecology and Obstetrics Congress in May 2009. This finding is similar to other publication ratios reported with regard to various national and international congress abstracts^(1,3,4,5)^. However, in these studies, only publications in international journals have been evaluated. Compared to these previous reports, a lower publication ratio has been found in our study for abstracts converted to publication in international journals. A lack in decent knowledge and skills needed to prepare a scientific article, an inadequate command over English language and grammer as well as the meticulous and demanding nature of the evaluation process of a manuscript submission in peer-reviewed and indexed journals in comparison to its counterpart in scientific congresses have been presumed as possible explanations for this low publication rate. On the other hand, a possible negative discrimination to studies reporting no statistically significiant difference or certain personal reasons, including lack of free time and motivation, might have been contributed to this fact as well.

Similar to the results of previous reports in the literature, the mean time to publication has been found as 11±4 months and 17±2 months in national and international journals respectively in our study^(6,7,8)^. Nevertheless, we believe that the publication time should be shortened so that the scientific articles provide more actual data when they are accessed by the readers of the journal. Considering the time interval dedicated to research and writing in addition to the time lost during the publication stage, it becomes evident that the findings presented in current articles have been actually established on average two years ago. On the other hand, the majority of the authors responsible for the conversion of the abstracts to publications have clinical duties and therefore they have a very limited time to spend for their academical activities. There are also other factors which result in a further lengthening of the total time necessary for a presentation’s conversion to publication. Considering the evaluation stage and revision requests frequently observed in national as well as international peer-reviewed journals, it takes some months for a meeting abstract to be published on average. Morever, many studies are accepted for publication after several previous submissions eventually rejected by other journals; a fact which further increases the total duration of time spent for publication.

Seven of the abstracts presented in the congress (2.8%) had been published previously in a peer-reviewed journal. Such a low ratio per se is a desired outcome for scientific congresses. The scientific evaluation committees usually tend to prefer previously unpublished research abstracts so that rather new studies and up-to-date items of information will be presented. On the other hand, the ratio of presentations that had been published before exhibits an obvious increase in recent years. In order to minimalize the risk of plagiarism, the researchers prefer to present their studies after being published as a journal article^(9)^.

Although statistically nonsignificant, the conversion rate of congress abstracts to publication has been found to be higher for presentations originating from university hospitals. This finding can be explained with a relatively higher level of motivation and academical goals of the university-based researchers who also are responsible of the education of medical school students and resident training. One of the primary focus targets for these researchers is the conversion of their studies to scientific publications in prestigious international and national journals. For this reason, the relatively higher rate of conversion is an anticipated finding for the university-based studies.

The meeting abstracts submitted to the National Gynecology and Obstetrics Congress can be presented either as an oral presentation or as a poster. Among the abstracts accepted for presentation by the evaluation committee, oral presentations are usually selected as those studies which promise to yield some of the greatest contribution scientifically. Therefore, it is not suprising at all to find out that the publication rate for the oral presentations in our study were significantly higher than the rate calculated for the posters. There are also other studies reported in the literature with a similar finding^(10,11,12,13,14)^. Nevertheless, some other scientific meetings also exist where very similar conversion rates had been observed for oral and poster presentations^(15,16,17)^. Although statistically not significant, the mean time to publication for oral presentations has been found to be almost 2 months shorter than that of the posters. These findings show that in comparison to posters, oral presentations have a relatively higher overall quality thanks to their metodology, applicability and data content.

In the publications, an alteration in author names has been encountered in 72.6% of congress abstracts. Similar findings have been reported in other studies in the literature^(1)^. The conversion of a meeting abstract to a scientific article in a journal is a challenging process requiring a great deal of hard work. Therefore, the names of new researchers are usually added to the authors list when they have made a significant contribution to the conversion of an abstract to publication. On the other hand, in order to increase the overall attendance to the congress and to enhance the motivation of young researchers, residents having only a limited contribution to the meeting abstracts have their names added to the authors list very frequently; some of them are even given the privilege of making the oral presentation personally. However, during the preparation of the manuscript, the names of those researchers, who had a very limited contribution to the execution of the study and to the writing phase, are excluded. Still other opinions exist on this subject, which suggest that a meeting abstract should be regarded as a publication by itself and it can be cited as a reference in scientific articles; therefore, the alteration in the authors list in any way should be regarded as a wrong act according to the authorship criteria set forth by the International Committee of Medical Journal Editors (ICMJE)^(18)^.

The present study had some limitations. A drawback of the present study could potentially arise from our search algorithm missing some papers due to a change in surname of female authors or due to a typing error in author names spelled in Turkish letters. Another restriction of our study was the databases used; papers published in different journals, which are not indexed in the databases utilized in this study, were not detected in our search.

## CONCLUSION

Although a significant part of the presentations held at the 2009 National Congress of Gynecology and Obstetrics were successfully published, only a limited proportion of these publications were in international journals. The conversion rate from meeting abstract to publication was found to be higher for oral presentations, which is obviously a fact reflecting the relatively higher scientific value of the studies accepted for oral presentations. The first step towards improving this overall low conversion rate should be the establishment of a solid notion for research as early as possible during the residency period. In order to accomplish this goal, faculty members, specialists and residents must be appropriately supported and the constitution of experienced research teams should be facilitated. In this way, we believe both the conversion rate of congress presentations to publications and the overall quality level of the scientific studies will be significantly improved. Apparently, new studies focusing on the aforementioned parameters are needed in order to prove the validity of this suggestion.

## Figures and Tables

**Table 1:**
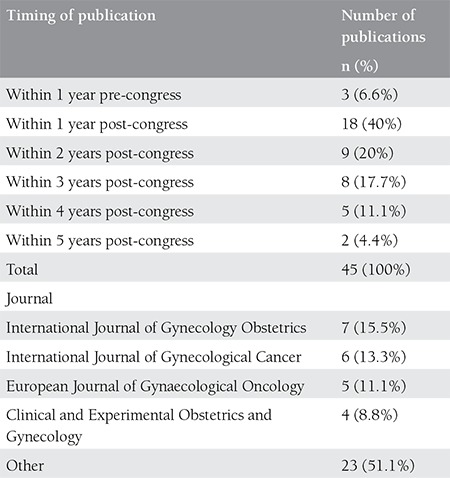
The distribution of the published presentations according to their timing of publication and the ranking of the international journals according to their content of published presentations

**Table 2:**
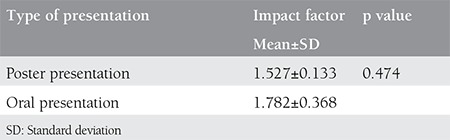
The comparison of journal’s impact factors according to the type of presentations which have been published

**Table 3:**
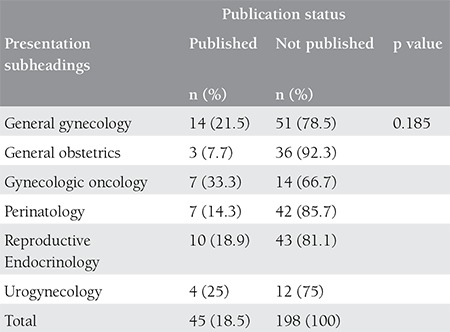
The comparison of publication rates in international journals according to various subheadings of presentation
